# Molecular dynamics study of lithium intercalation into –OH functionalized carbon nanotube bundle

**DOI:** 10.1038/s41598-022-13509-2

**Published:** 2022-06-14

**Authors:** Bin Zheng, Huaze Dong, Jinmiao Zhu, Yanping Wang

**Affiliations:** School of Chemistry and Chemical Engineering, Hefei Normal University, Hefei, 230061 Anhui People’s Republic of China

**Keywords:** Computational science, Nanoscience and technology

## Abstract

The influence of hydroxyl group (–OH) on carbon nanotube (CNT) interacting with lithium (Li) ions has been investigated via *ab initio* molecular dynamic (MD) simulations. Compared with the pure CNT, a higher efficiency has been observed for lithium intercalating into CNT-OH bundle. At lower Li ion density and CNT bundle density, CNT-OH exhibits higher intercalation efficiency than the pristine and ammonium functionalized CNTs. As the increasing of Li ion densities and CNT bundle densities, Li ions tend to intercalate into the interlayer between CNT-OH tubes instead of the interior of CNT-OH tubes. We also observe the destruction of hydroxyl groups during the intercalation of Li ions into interlayer of CNT-OH bundle. It is therefore suggested that eliminating the intercalation of Li ions into interlayer between tubes is important for the design of Li ion batteries.

## Introduction

Since traditional fuel energy is limited and overexploited, the burst of global warming urged scientists to find the renewable or new kinds of energy forms^1^. As for the applications of portable devices or vehicles, materials with excellent charging and recharging performances are in increasing demand. Among them, Li ion batteries (LIBs) with advantages of excellent cyclic stability, high capacity density and good energy storage have shown tremendous applications fields in daily-life. As the performances of energy transfer rely on materials adopted, utilization of nanotechnology to construct tunable and designable nano-materials with desirable characters becomes incremental important.

Due to the atomic structure and surface modification characters, carbon based nano-materials have attracted much attention for energy conversion and storage. Since their findings in early 1990s, CNTs have been recommended in diverse applications^2,3^. They have many fascinating performances such as excellent electronic sensitivity, good mechanical strength, high surface area to volume ratio, good electronic sensitivities, high flexibility and extraordinarily electro-catalytic activities which endow them with practical applications in sensors, actuators, energy storage equipment, et al.^4,5^.

Although significant progress has been made, the problem of solubility hampers further applications. Pristine CNTs are insoluble in water or organic solvents, and have strong tendencies to aggregate into bundles owing to the strong π-bonds on CNTs’ surfaces. These strong covalent bonds lead to heterogeneous distribution and limit the application of CNTs in industrial scale. To fabricate efficient electrode, CNTs are treated either by covalent modification of chemical groups or non-covalent attachment of functional molecules^6,7^. For electro-catalytic application, CNTs should be pre-treated before usage. The air oxidation^8^ and mixed acid^9^ methods have been applied to confer CNT with oxygen-containing groups such as carboxylic or hydroxyl groups on the surface^10^ with which the exfoliation of bundles can be alleviated and homogeneous dispersion can be enhanced. For pure CNTs, the simultaneous satisfaction of high energy density and power density is difficult to realize since the storage mechanism depends on a physical intercalation process. The doping as well as modification have been applied and excellent outcomes have been achieved^11,12^. It was reported that the defected CNTs could attain a reversible capacity of 650 mA h/g^13,14^, and the lithium insertion for CNTs can cover a range of 220–780 mA h/g^15^. Except for the solubility, high columbic efficiency is another key problem. Some research concentrated on the morphological characters of CNTs^16–20^. The mechanism of charge transfer between Li and deformed CNTs are also in great concern. It was found that lithium intercalation may lead to CNTs deformation^17^ and surface adsorption mechanism was also investigated for the process^18^.

To solve this simple yet key problem, studies should be conducted. Experiments are direct but time consuming and expensive. Simulation can thus be an alternative, guiding and complementary way. The first principle method showed that in 1 M LiClO_4_ EC/DEC, the lithium intercalation occurred at the closed ends exhibiting a reversible capacity of 125 mA h/g^19^. Utilizing density functional theory (DFT) calculations, the diffusion of Li in CNT armchair (5,5) from radial and axial directions was reported^20^ and the lithium insertion capacities were reported to be dependent on chirality^21^. The influence of chirality on lithium interaction with CNTs was also calculated based on DFT^22,23^. It was shown that Li could quickly penetrate into CNTs or exist between neighboring CNTs. Defects in the side walls of CNTs presented easy diffusion of Li ions^24,25^. Ab initio study was used to study lithium intercalation into CNTs with amine and carboxyl groups (NH_2_/((8,0)) and COOH/((8,0)), and shows more preferential intercalation tendencies compared with pristine CNTs^26^. Different functional groups onto CNTs have been studied and it was inferred that the introduction of functional groups would induce the transition from semi-metal to semiconductor status^27^. The intercalation and diffusion of Li ions in CNTs was first studied by Fang’s group^28^ and effects of functional group on Li ion crossing CNTs as well as the enhanced intercalation of Li into CNTs by amine functionalization were also investigated^29,30^. Other influences such as electrolytes and van der Waals (vdW) interaction on the process were studied^31–33^.

Although several outcomes were achieved, the information is still limited. All the conclusions from simulations were drawn with static states while the actual situations are dynamic processes. Therefore, the evaluation during a dynamic process is in great need. To evaluate the potential application of functional groups on Li ion intercalation in kinetic, in this work we investigated the hydroxyl group functionalized CNTs from axial and radial directions with different lithium ions densities and CNTs bundles densities.

## Computational methods

Single walled CNTs (SWCNT) with chirality (8,0) and saturated hydrogen atoms on each ends were constructed in this work. They have zigzag conformations with 128 carbon atoms in total. Seven repeat units were arrayed along the tube axis and the size was set at a length of 17.84 Å and a diameter of 3.19 Å. The hydroxyl group functionalized CNTs (CNTs-OH) were formed by chemisorbing 24 hydroxyl groups at the side walls of CNT and four hydroxyl groups were averagely distributed at two ends. The origin of the coordinate system was set the same as pristine CNTs and CNT-NH_2_ located at the center of the nanotube referred to our previous work^30^. The ends of the CNTs were initially set at z = 8.92 Å and − 8.92 Å. Two different Li ion densities were studied for lithium intercalation utilizing MD simulations.

For low Li density study, eight Li ions were set at the upper and bottom ends of CNTs. Their coordinates were set at (0.85, 1.47, z_0_), (0.85, 1.47, z_0_), (5.20, 3.00, z_0_) and (5.20, 3.00, z_0_), where z_0_ = 13.0 or − 13.0 Å. To exclude the influence of Li ion with CNTs’ interactions at the beginning of simulation, the distance between lithium and CNT end edge was set to be 4.1 Å. For high Li ion density study, twenty lithium atoms were added into the system. Aside from previous eight Li ions, additional twelve ions were set at (3.90, 2.25, z_0_), (3.90, 2.25, z_0_), (6.06, 3.50, z_0_), (6.06, 3.50, z_0_), (5.00, 0.00, z_0_), (0.00, 5.00, z_0_), where z_0_ = 13.0 or − 13.0 Å. To make the study more specific, two confined hexagonal boxes with dimensions of 13.0 × 13.0 × 32.0 Å^3^ and 15.0 × 15.0 × 32.0 Å^3^ were adopted for the distance investigations of CNT-OH interacting with Li.

Visual Molecular Dynamic (VMD)^35^ was utilized to create the initial structure of CNT-OH. The initial conformation with or without the addition of Li were submitted for further calculations using Car–Parrinello MD simulations. In this work, Car–Parrinello MD^36^ was performed using the CPMD software package^34^. In all calculations, Perdew, Burke, and Ernzerhof (PBE) exchange–correlation functional^37,38^ was used. To assure energy convergence, a plane wave set with 80 Ry kinetic energy cutoff was adopted for valence electrons. The Troullier–Martins normconserving nonlocal pseudopotential^39^ was utilized to describe the valence electrons and ionic cores interactions in the Kleinman–Bylander form^40^. The preconditioned conjugate-gradients (PCG) method and the L-BFGS method^41^ were utilized for wave function optimization and geometry calculation. In the MD calculations, the temperature was set to 300 K. The massive Nose–Hoover chain algorithm^41,42^ were utilized to achieve thermostatting. All the hydrogen atoms were substituted by deuterium atoms, and a time step of 4.0 a.u. (0.09676 fs) and a fictitious electronic mass of 500 a.u were used. At the beginning of MD simulations, the electronic wave functions were quenched to the Born–Oppenheimer surface. The kinetics study lasted 20 ps for CNT-OH system, and trajectories were collected every five steps. Periodic boundary conditions (PBC) are applied in all directions to simulate a bundle of CNTs, and only the Ʈ point of the Brillouin zone was considered for periodically repeated system calculation.

In the present work, all the calculations for the intercalation of Li ions into the functionalized CNT bundles are performed in gas phase without the inclusion of solution environments. To be noted is that this setup is somewhat different from experiment. In experiment, electrolytes are necessary for solution phase. However, solution phase is not considered during modeling due to the massive computational power required for ab initio MD calculations at the electronic structure level. Therefore, unsolvated lithium only in gas phase is discussed.

## Results and discussion

**Figure 1:**
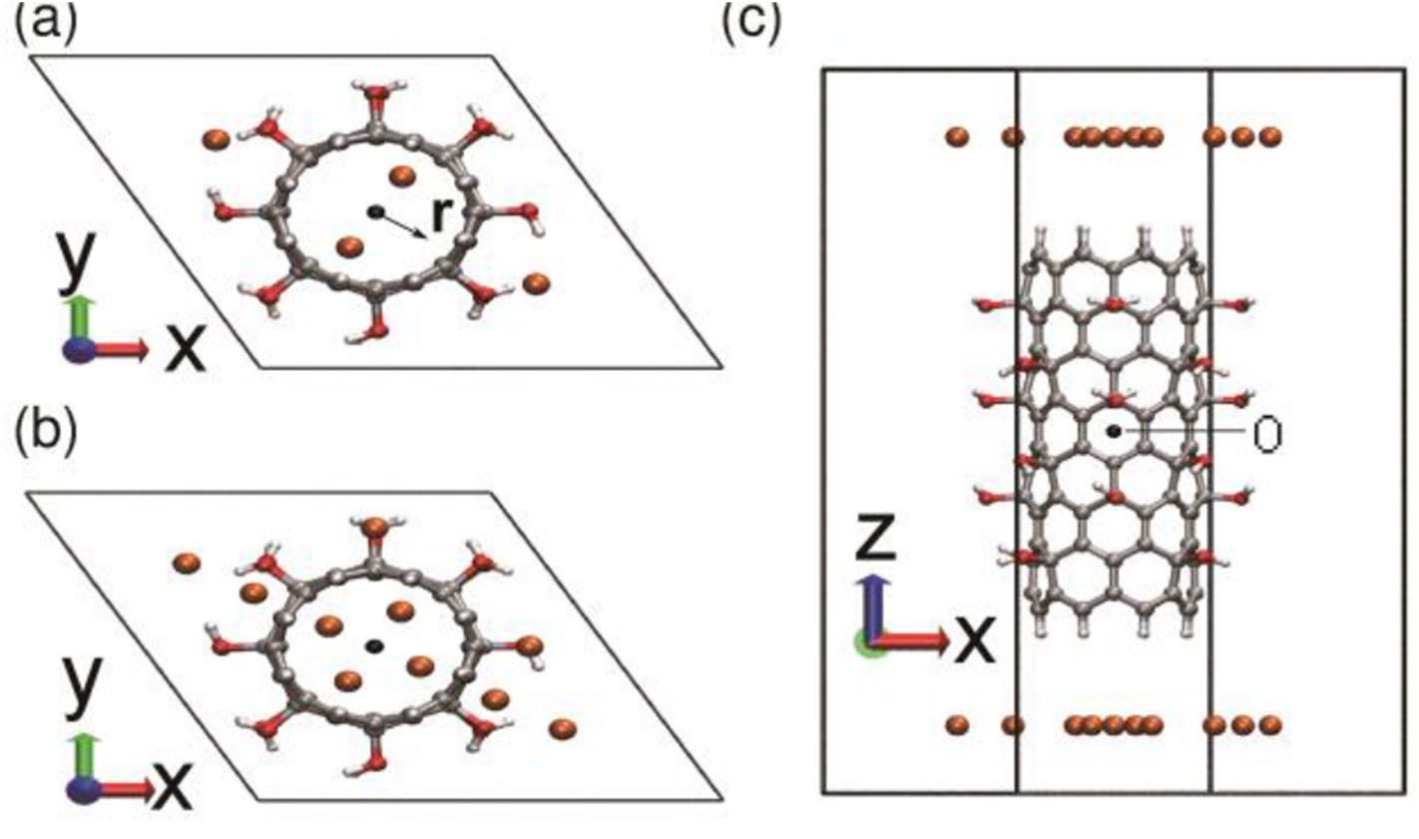
Initial conformation of the unit cell for* ab initio* MD simulations. The lithium and hydrogen atoms are shown in orange and light gray, respectively; the carbon atoms are displayed in dark gray; and the oxygen atoms are in red. (**a**) and (**b**) are the –OH functionalized CNT with four and ten lithium atoms placed upon each end of the CNT, respectively. (**a**,**b**) Top view; (**c**) side view. The symbol O indicates the origin of the coordinates. The radial distance between the lithium atoms from the central axis of the nanotube is denoted by the symbol r used together with the black arrow. The pictures are generated by VMD 1.8.3 software. URL link: http://www.ks.uiuc.edu/Research/vmd/.

The stability of CNT-OH was first determined by geometry optimization calculations. As calculated in previous work^29^, the C–C bond distance in pristine CNT is 1.42 Å while for CNT-OH, a small distortion induced by the polar groups, leading to a bond length of 1.52 Å. The bond length of the C–O bond is 1.47 Å. The starting conformation of Li ions with CNTs was shown in Fig. 1. 20 ps simulations of CNT-OHs with Li ions were performed using *ab initio* MD simulations.

### Lithium intercalation into CNT bundles at low Li ion density

**Figure 2:**
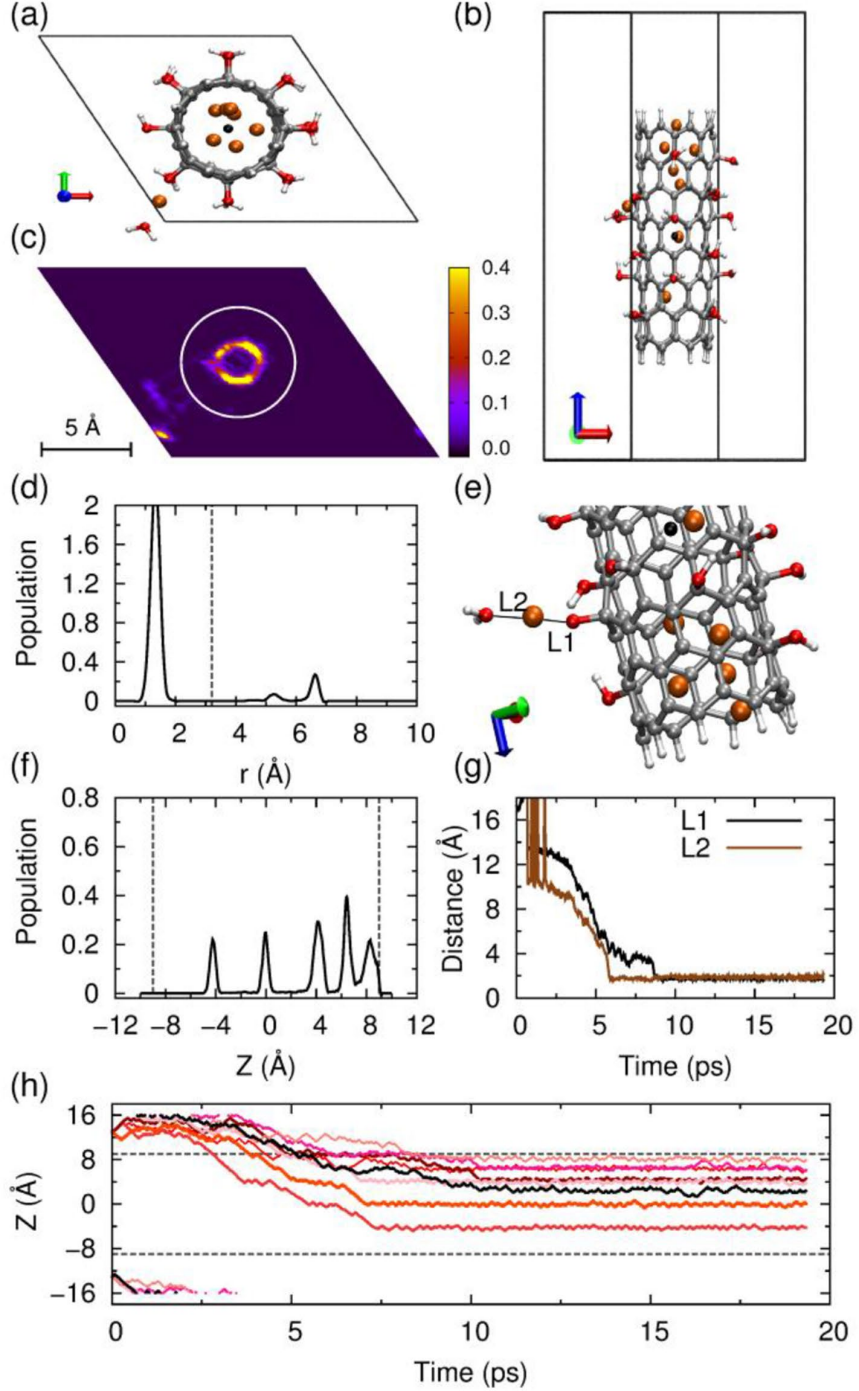
Behavior of the eight Li ions intercalating and diffusing into the bundle of –OH functionalized CNTs. The system was simulated in a hexagonal box with dimensions of 15.0 × 15.0 × 32.0 Å^3^. (**a**) Typical snapshot of the unit cell, view from the top. (**b**) Side view. (**c**) Distribution of the Li ions in the x–y plane. (**d**) Radial distribution of the Li ions, and the vertical dashed line represents the wall of nanotube. (**f**) Axial distribution of the ions within CNT, only calculated for the ions within the nanotube. The two ends of CNT are indicated by the two vertical dashed lines. (**e**) A snapshot for structure of the –OH groups and the Li ion located at the space between the nanotubes, and the distance between lithium and oxygen atoms are displayed in (**g**). (**h**) The z-position as a function of simulation time (ps) for each Li ion. The upper and lower dashed gray horizontal lines denote the top and bottom ends of CNT, respectively. The pictures of (**a**–**c**,**e**) are generated by VMD 1.8.3 software. URL link: http://www.ks.uiuc.edu/Research/vmd/. The plots of (**d**,**f**–**h**) are generated by gnuplot 5.0 software. URL link: http://www.gnuplot.info/.

We first simulated the system with a low Li ion density. A low bundle density with a hexagonal box with dimensions of 15.0 × 15.0 × 32.0 Å^3^ is used, and eight Li ions were placed at the ends of CNT-OH bundle, as shown in Fig. 1c. Z coordinate denotes the height of the CNT-OH, whose center is set to zero. The two dashed lines represent the upper and lower boundaries of CNT-OH. As shown in Fig. 2h, half of the eight Li ions first distribute evenly at the upper side and the lower side. Then they sequentially diffuse into the interior or the interstitial channel of CNT-OH from the upper part of the CNT-OH. It is shown that all ions locate in the range from − 8.9 to 8.9 Å, indicating the successful lithium intercalations into CNT-OH bundle. The monitoring of Z for each Li ion indicates that the fastest Li ion enters the CNT-OH tube at 3 ps, and the slowest one at 8 ps. As shown in Fig. 2f, the distribution of ions in Z suggests that most of the Li ions locate at the upper part of the bundle, and only one locate at lower part. More importantly, the sharp peaks in the distribution profile suggest that the adhesions of Li ions onto CNT-OH tube are stable and cannot be easily broken at room temperature.

The radial distribution of Li ions on the x–y axis and snapshot are shown in Fig. 2d,c, respectively. As presented in Fig. 2a, seven of the total eight Li ions enter into the CNT-OH interior, and one moves into the channel between neighboring CNT-OH tubes. The Li ions stay on a cylindrical surface with a radius of 1.4 Å and distribute almost evenly within CNT-OH. Detailed inspections into the behaviors of the Li ion in the interstitial channel process are shown in Fig. 2e,g. The only one Li ion in the channel area first diffuses among tubes, and then connects with the surface hydroxyl group, and finally leads to the destruction of hydroxyl groups from the CNT-OH. This process is spontaneous and happens within 6–9 ps. A side view of system after Li ion intercalation is presented in Fig. 2b.

**Figure 3:**
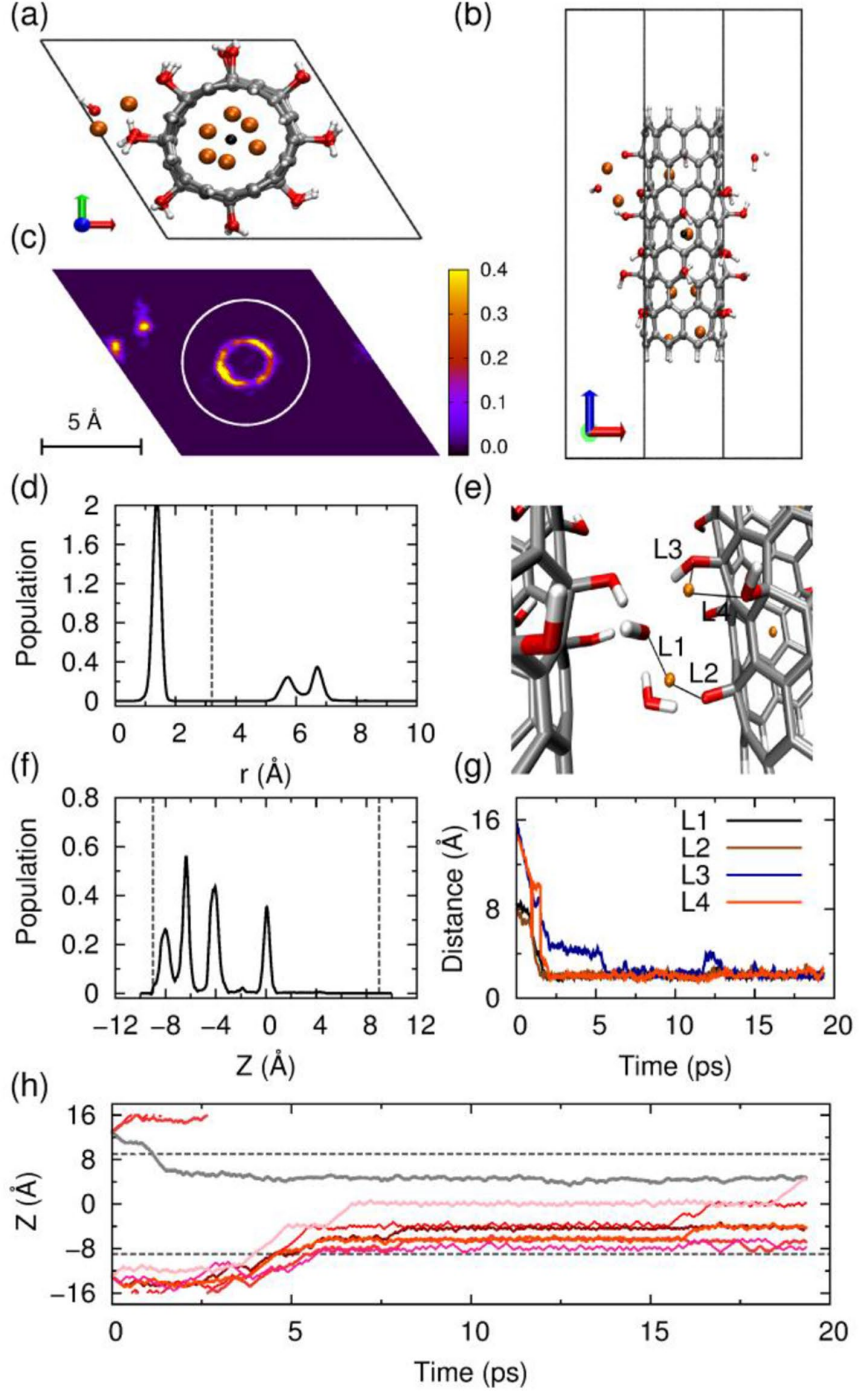
Behavior of the eight Li ions intercalating and diffusing into the bundle of –OH functionalized CNTs. The system was simulated in a hexagonal box with dimensions of 13.0 × 13.0 × 32.0 Å^3^. (**a**) Typical snapshot of the unit cell, top view. (**b**) Side view. (**c**) Distribution of the Li ions in the x–y plane. (**d**) Radial distribution of the Li ions, and the vertical dashed line represents the wall of nanotube. (**f**) Axial distribution of the ions within CNT, only calculated for the ions within the nanotube. The two ends of CNT are indicated by the two vertical dashed lines. (**e**) A snapshot for structure of the –OH groups and the Li ion located at the space between the nanotubes, and the distance between lithium and oxygen atoms are displayed in (**g**). (**h**) The z-position as a function of simulation time (ps) for each Li ion. The upper and lower dashed gray horizontal lines denote the top and bottom ends of CNT, respectively. Because the simulation is performed under periodic boundary condition, these ions that move down towards the bottom edge of the simulation box will simultaneously appear at the top edge of the box. The pictures of (**a**–**c**,**e**) are generated by VMD 1.8.3 software. URL link: http://www.ks.uiuc.edu/Research/vmd/. The plots of (**d**, **f**–**h**) are generated by gnuplot 5.0 software. URL link: http://www.gnuplot.info/.

To further validate the impact of Li ions on the stabilities of hydroxyl groups, a hexagonal box with dimensions of 13.0 × 13.0 × 32.0 Å^3^ was constructed. The smaller box dimensions give closer distances between CNT bundles. As presented in Fig. 3a, six of the total eight Li ions enter into the CNT-OH interior, and two move into the channel between neighboring CNT-OH tubes. The vivid image for Li ions contribution in axial direction is shown in Fig. 3c. The MD simulations show that six Li ions diffuse into the CNT interior and present a cylindrical distribution along the central axis and a sharp distribution alongside the axis as shown in Fig. 3d,f. The left two Li ions move into the bundle interlayers with distances of 2.1 and 3.7 Å to the CNT sidewalls. The snapshot of the interlayer situation is shown in Fig. 3e, and a side view of system after lithium intercalation is presented in Fig. 3b. Careful examinations of the behaviors of Li ion locating in the interlayers between CNT-OH tubes show that Li ions can quickly interact with the hydroxyl groups on CNT within 2 ps. The binding of Li ions with –OH groups induces the dissociations of –OH groups from CNT-OH surface. More interestingly, the released hydroxyl groups can even form a water molecule, as shown in Fig. 3e. This process occurs within 2 ps as indicated from Fig. 3g.

The z-positions as a function of simulation time (ps) for each Li ion (Fig. 3f) show that Li ions tend to concentrate at the bottom of the CNTs, and only one ion moves into the upper part of CNT-OH. The first Li ion enters into the CNT-OH bundle through diffusion in 1 ps, and stays at Z ≈ 0 Å during our 20 ps MD simulation. Five of the eight ions diffuse into the CNT-OH interior sequentially at a time scale from 4 to 5.5 ps, and distribute in the range from Z ≈ − 4 Å to Z ≈ − 8 Å. In this system, 75% Li ions move into the interior of CNT-OH while 25% stay in the interlayers between tubes as obtained from Fig. 3h. Previous work shows that for pristine CNT, only 37.5% Li ions intercalate successfully while for ammonium functionalized system 87%^29^. Overall, the hydroxyl functionalized system with low Li ion density and low bundle density can achieve an intercalation efficiency of 87.5%. However, the lithium intercalation among interlayers leads the releasing and deactivation of –OH groups. Therefore, how to increase the lithium intercalation into interior of CNT is the key problem for hydroxyl functionalized CNT.

### Lithium intercalation into CNT bundles at high Li ion density

**Figure 4:**
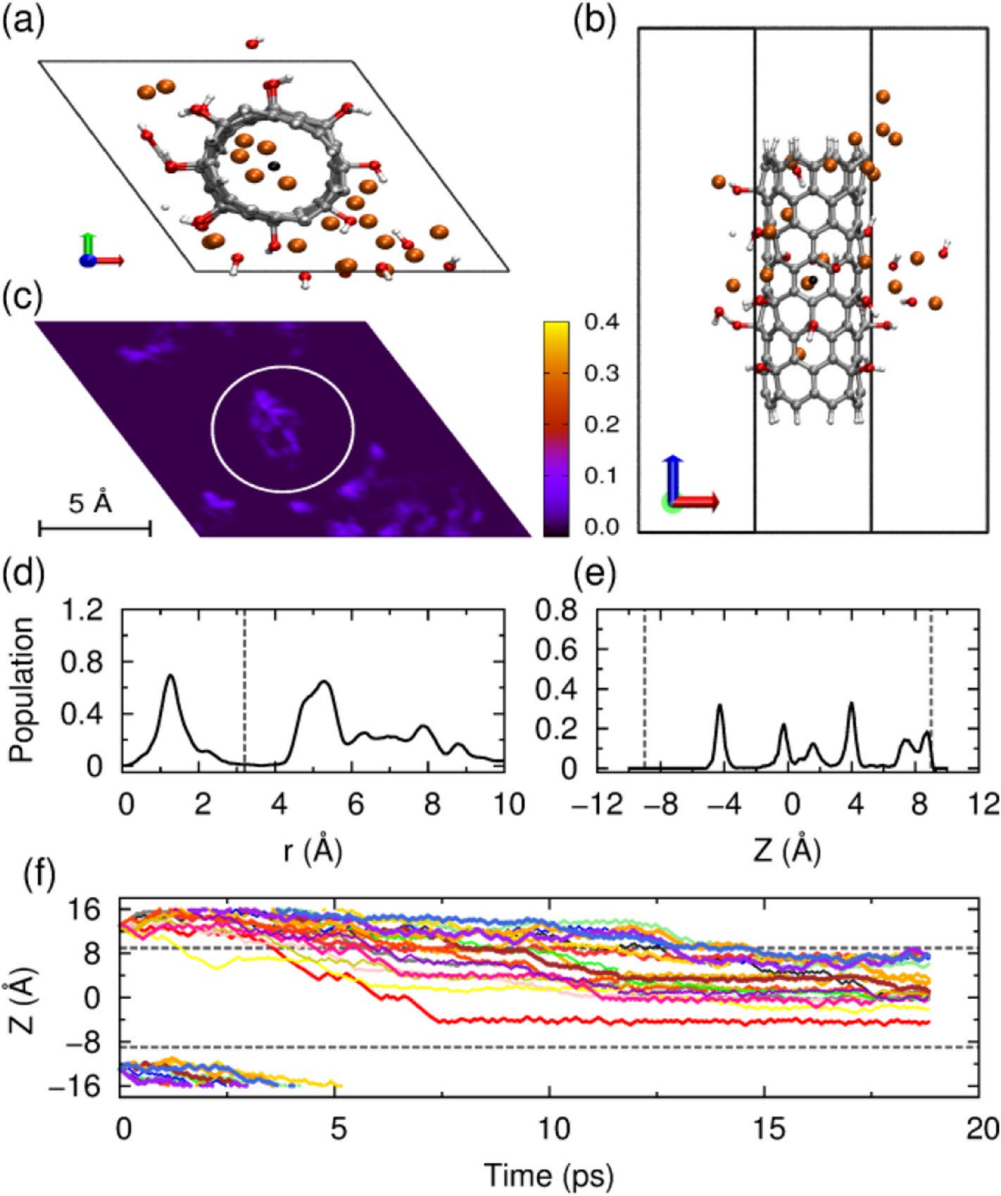
Behavior of the twenty Li ions intercalating and diffusing into the bundle of the –OH functionalized CNTs, simulated in a hexagonal box with dimensions of 15.0 × 15.0 × 32.0 Å^3^. (**a**) Typical snapshot of the unit cell, top view. (**b**) Side view. (**c**) Distribution of the Li ions in the x–y plane. (**d**) Radial distribution of the Li ions. (**e**) Axial distribution of the ions within CNT. (f) The z-position as a function of simulation time (ps) for each Li ion. The pictures of (**a**–**c**) are generated by VMD 1.8.3 software. URL link: http://www.ks.uiuc.edu/Research/vmd/. The plots of (**d**–**f**) are generated by gnuplot 5.0 software. URL link: http://www.gnuplot.info/.

Herein, a high lithium ion density of twenty Li ions was conducted. We first simulated a hexagonal box with dimensions of 15.0 × 15.0 × 32.0 Å^3^. As shown in Fig. 4a,b, five Li ions diffuse into the interior of CNTs while the left ones swim in interlayers between the CNT-OH. With the increasing of free Li ions, more hydroxyl groups dissociate and more water molecules form (Fig. 4b). The snapshot picture and radial distribution in Fig. 4c,d, exhibiting dense arrangements of Li ions in the CNT interior. Although the peak is not as sharp as low Li ion density, the arrangement is relative concentrated. Li ions outside of the CNT sidewalls present different and asymmetric distribution. As for the radial distribution, Li ions tend to arrange in the middle and upper sides outside of CNTs. Inside of the CNTs, Li ions distributed relatively symmetric, with two ions locating near the upper end of CNTs as presented in Fig. 4d,e. Similar to the case of low Li ion density with larger box, Li ions moves into CNT-OH from the upper end. At first, seven of twenty ions vibrate and flow to the lower end and then back to the upper end, in a sequential way. The first one arrives at the CNT-OH end in 1 ps, as shown in the bright yellow line in Fig. 4f. The second ion achieves its intercalation at ~ 4 ps, and all the other ions achieves the intercalation successively in ~ 13 ps and stabilize at 12–15 ps. This intercalation way is similar to the ammonium functionalized CNTs but is faster and the distribution is more concentrated. 25% of Li ions intercalated into the interior of CNT-OH while only 15% for ammonium functionalized CNT^29^. Since Li ions act as dissociation catalysis for –OH groups, the more ions locating at the CNT-OH interior implies less damage and dissociation to the functional groups.

**Figure 5:**
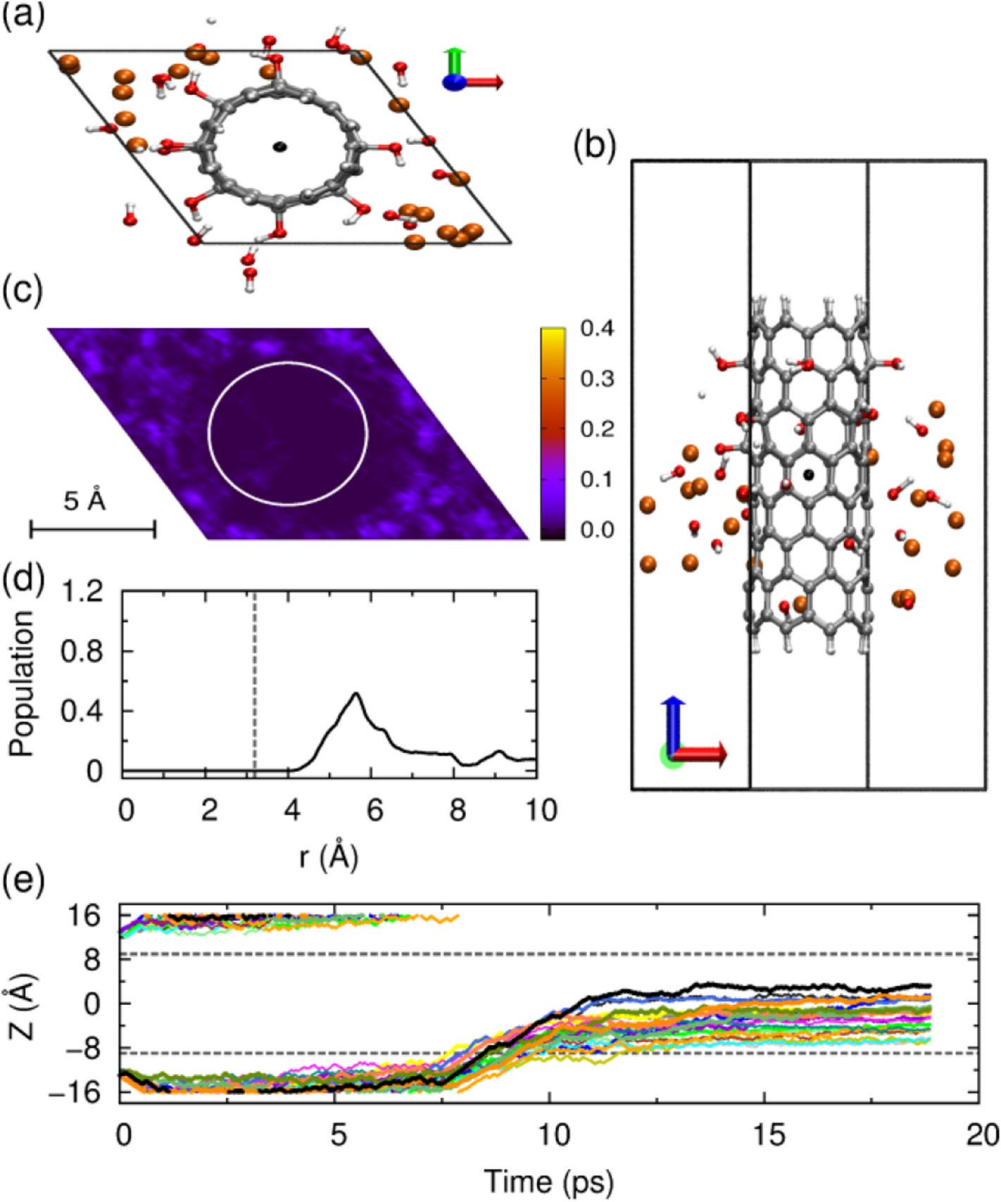
Behavior of the twenty Li ions intercalating and diffusing inside or between –OH functionalized CNTs. The system was simulated in a hexagonal box with dimensions of 13.0 × 13.0 × 32.0 Å^3^. (**a**) Typical snapshot of the unit cell, view from the top. (**b**) Side view. (**c**) Distribution of the Li ions in the x–y plane. (**d**) Radial distribution of the Li ions and the vertical dashed line represents the wall of nanotube. (**e**) The z-position as a function of simulation time (ps) for each Li ion. The upper and lower dashed gray horizontal lines denote the top and bottom ends of CNT, respectively. The pictures of (**a**–**c**) are generated by VMD 1.8.3 software. URL link: http://www.ks.uiuc.edu/Research/vmd/. The plots of (**d**,**e**) are generated by gnuplot 5.0 software. URL link: http://www.gnuplot.info/.

High bundle density with hexagonal box dimensions of 13.0 × 13.0 × 32.0 Å^3^ was also investigated. As depicted in Fig. 5a, none of the Li ions diffuse into the interior of CNT-OH, and they all disperse randomly around CNTs interlayers instead of interior. More released hydroxyl groups or formed water molecules are observed from the side view in Fig. 5b. The snapshot and radial population in Fig. 5c,d validate that none of Li ions reach the interior space. Our examinations indicate that five of the twenty Li ions move upwards while the left all vibrate from the bottom end into the lower or center part shown in Fig. 5e. Compared with low bundle density, higher bundle density will screw more Li ions into the gaps between CNTs, resulting in the dissociation of more functional groups. These phenomena suggest that a high bundle density along with a high Li ion concentration is unsuitable for lithium intercalation.

Although CNT structure is symmetric, the final lithium distribution is asymmetric in the Z axis for different lithium densities. This may due to the following two reasons. The simulation is conducted at 300 K which is a relative low temperature. At this temperature, the diffusion of Li ions in CNT is slow. For another, the timescale for our investigation is within 20 ps. This timescale is large for simulation but relatively small for experiments. Therefore, it is not long enough for Li ions to distribute evenly as expected.

### Energetics of lithium intercalation into CNT-OH

To investigate the mechanism of intercalation, energetics of the intercalation processes were calculated to evaluate the tendency of different pathways. Before calculations, CNT-OH bundles were firstly optimized. After placing Li ions at specified positions, the intercalation energy E_ad_ is calculated as, E_ad_ = E_1_ − E_0_, where E_1_ denotes the energy of the system when Li ion locates in the interior of CNTs, and E_0_ representing the zero point energy of the system when Li ions are placed at z = 11 Å. This is approximately 2.0 Å from the top end of tubes.

**Figure 6:**
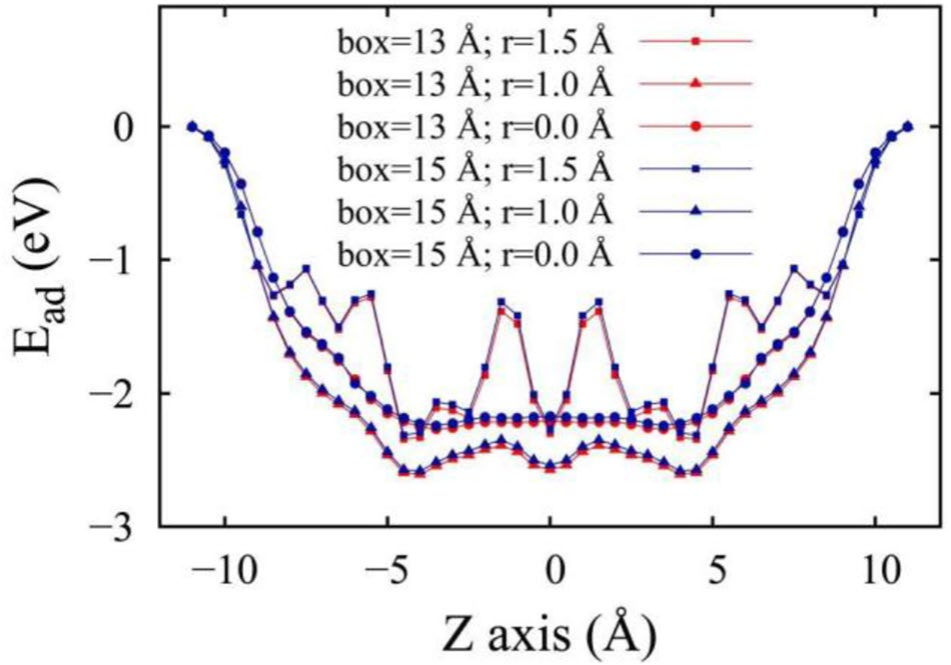
Intercalation energy of a lithium atom within the –OH functionalized CNT. The lithium atoms is placed at position z and in the interior at r = 1.5 Å, 1.0 Å and 0.0 Å. The two ends of the nanotube are indicated by the vertical dashed lines. This figure is generated by gnuplot 5.0 software. URL link: http://www.gnuplot.info/.

The influences of different bundle densities and radial distances are depicted in Fig. 6. At the same radial distances, the bundle density affects the shapes of the E_ad_–Z curve negligibly. Careful examinations indicate that the blue curves are slightly higher than the red curves, suggesting a denser CNT-OH bundle gives lower intercalation energies. CNT-OH bundles in boxes sized 15.0 × 15.0 × 32.0 Å^3^ and 13.0 × 13.0 × 32.0 Å^3^ with the radius 0.0 and 1.0 Å present similar outlines as pristine CNTs and ammonium functionalized CNTs^29^. Two potential wells locate at z equals 4.5 and − 4.5 Å. For the case of r = 1.0 Å, a third potential well appears at z = 0 Å. The shapes of r = 1.5 Å are more complicated presenting zigzag curves and show additional apexes at Z points of 1, 1.5, − 1, − 1.5 Å which are in accordance with above figures.

## Conclusions

The effects of hydroxyl group modification on the interaction of Li ions into CNT-OH bundle have been studied using *ab initio* MD simulation method. It is demonstrated that Li ion can intercalate into the interior and interlayer of CNT-OH bundle. The effects of lithium ions density and bundle density on functional group changes have been carefully investigated and the dynamic processes have also been monitored.

Lower Li ions density with low bundle density is efficient for the intercalation of Li ions, with its efficiency as high as 87.5%, which is higher than the reported pristine and ammonium functionalized CNTs. However, our calculations show that the Li ion can intercalate into the interlayers and the interiors. For the Li ions locating at the interlayer, they can catalyze the dissociation of hydroxyl functional group from the CNT-OH. Our results suggest that the intercalation into interiors of CNT-OH is more preferential for energy storage. Further energetic calculations show that denser densities of CNT bundles induce more dispersed Li ions in the CNT-OH interlayers, it is therefore suggested that eliminating the interlayer intercalation will be more applicable for rechargeable LIBs.

Lastly, the limitations of the present study are discussed. VdW interactions are important in layered systems, although slight difference between the electronic structure properties for the results calculated using PBE functional with vdW corrections and that using PBE functional without vdW interactions^43^. Besides, the influences of electrolytes are not within consideration and only chirality of (8,0) has been investigated. Therefore, future work with full considerations of vdW, electrolyte and chirality influences is suggested for better fitting with practical applications.

## Data Availability

All data generated or analyzed during this study are included in this published article.
